# The impact of discrimination perception on academic burnout of junior college students: the mediating role of belief in a just world and the moderating effect of sense of family obligation

**DOI:** 10.3389/fpsyg.2026.1732040

**Published:** 2026-02-18

**Authors:** Wenjing Du, Qi Yan, Mengli Zhang, Xiaomin Lv, Maocong Zhang

**Affiliations:** School of Public Management, Shandong Normal University, Jinan, China

**Keywords:** academic burnout, belief in a just world, discrimination perception, family obligation, junior college students

## Abstract

Within the context of higher education stratification, junior college students are more likely to experience unfair treatment based on their academic credentials during their studies and in societal evaluations. This social pressure may impact their academic engagement, educational quality, and long-term development. Based on the student needs-resources process framework, this study views academic burnout as a process outcome gradually formed under the dual pressures of continuous learning demands and resource depletion. Using structural equation modeling, it examines the relationships and underlying mechanisms among perceived of discrimination, belief in a just world, sense of family obligation, and academic burnout among junior college students from the perspectives of social structural pressures and their psychological mechanisms. The research results show that perception of discrimination has a significant positive predictive effect on academic burnout. Belief in a just world plays a partial mediating role between perception of discrimination and academic burnout. Among them, perception of discrimination significantly negatively predicts belief in a just world, and belief in a just world significantly negatively predicts academic burnout. Furthermore, sense of family obligation exerted a significant negative moderating effect on the relationship between perceived discrimination and academic burnout, while also negatively moderating the relationship between perceived discrimination and the belief in a just world. This study, framed within the demand-resource and meaning systems perspectives, elucidates the psychological mechanisms through which perceived discrimination influences academic burnout among junior college students. It provides empirical evidence for understanding how to enhance educational quality and promote students’ holistic development within stratified educational contexts.

## 1 Introduction

Academic burnout, characterized as a persistent psychological state marked by emotional exhaustion, learning apathy, and a diminished sense of accomplishment, has been demonstrated to be closely associated with reduced academic engagement, increased risk of dropping out, and mental health issues (Maslach et al., 2001; Madigan and Curran, 2021). Academic burnout, apart from emotional exhaustion, also encompasses elements of meaning such as cynicism and estrangement towards school and the significance of learning, as well as a sense of powerlessness, that is, the denial and indifference towards the value of learning (Salmela-Aro et al., 2009; Fajardo-Bullón et al., 2020). Over the past decade, research on academic burnout among college students has deepened both theoretically and methodologically. Research perspectives have gradually shifted from focusing on isolated emotional responses toward a process-oriented framework centered on the imbalance between “learning demands and resources.” This framework emphasizes the independent and interactive effects of multiple demands and resource allocations within learning contexts on the formation and developmental trajectories of student burnout (Salmela-Aro and Upadyaya, 2014). Meanwhile, meta-analyses and longitudinal studies indicate that academic burnout is not a transient emotional fluctuation but a developmental state that gradually accumulates during the learning process, correlating with subsequent academic outcomes (Bask and Salmela-Aro, 2013; Turhan et al., 2023). Consequently, some research draws upon the Job/Study Demands–Resources model to conceptualize academic burnout as a process-oriented outcome that progressively emerges within the context of sustained learning demands and resource depletion (Bakker and Mostert, 2024).

As of 2024, there were a total of 38.9126 million students enrolled in regular and vocational undergraduate and junior college programs in China, among which 17.6466 million were junior college students (Ministry of Education of the People’s Republic of China, 2025). Compared with undergraduates, junior college students face more structural challenges in terms of learning resources, social evaluation and development expectations (Ministry of Education of the People’s Republic of China, 2022). Particularly within China’s stratified higher education system, junior college students—as a distinctly labeled educational cohort—are more susceptible to experiencing pressure and negative judgments based on their academic credentials during social evaluation and resource acquisition processes (China Youth Net, 2025). Consequently, they are more susceptible to academic burnout. Existing research on academic burnout predominantly focuses on learning context factors and individual psychological characteristics, with insufficient attention to pressure sources from broader societal levels—particularly the experiences of social evaluation and identity devaluation faced by students within the context of educational stratification. Consequently, the formation of academic burnout is no longer solely a matter of learning contexts or individual traits; it must be understood from the perspective of how social factors influence academic adaptation through individual psychological processes. Among these, perceived discrimination—the subjective experience of unfair treatment based on educational background—emerges as a significant structural stressor potentially playing a pivotal role in the development of academic burnout among junior college students.

While existing research has examined the relationship between perceived discrimination and academic burnout, revealing a significant correlation, however, most studies treat discrimination as a general stressor, employing more universal explanatory frameworks such as negative emotions or psychological distress to account for its connection to academic burnout (Williams-York et al., 2024). Studies involving junior college students also frequently present pathways such as “perceived discrimination—negative emotions—academic burnout” (Li et al., 2024), and more specific psychological mechanisms need to be introduced. The core of academic burnout is not merely emotional exhaustion, but also includes alienation from the meaning and value of learning (Salmela-Aro et al., 2009). The meaning construction model indicates that when persistent stress experiences conflict with an individual’s core beliefs, goals, and values, it is more likely to trigger impaired meaning and weaken adaptive behavior (Park, 2010). In educational settings, university students’ sense of life meaning exhibits a significant negative correlation with academic burnout, and this sense of meaning can mediate the relationship between other psychological factors and academic burnout (Jin et al., 2022). Simultaneously, as a core belief within the meaning system regarding “world order and deserved rewards for effort” (Lerner, 1980), the just-world belief necessitates examination of its mediating role in the influence mechanism of perceived discrimination on vocational students’ academic burnout. Considering that individual differences in family role expectations may affect responses to stressful events (Fuligni et al., 1999; Goode, 1960), and family-level value and process factors can moderate the association between daily discrimination and distress responses (Valentino et al., 2025). Therefore, this study further examines the moderating role of family responsibility in the “perceived discrimination—belief in a just world—academic burnout” mechanism.

## 2 Theoretical basis and research hypotheses

This study adopts the student needs-resources process framework as its overarching theoretical perspective, conceptualizing academic burnout as a process-oriented outcome that gradually emerges within the context of sustained learning demands and resource depletion. Within this framework, perceived discrimination is regarded as an additional social learning demand that may increase the risk of academic burnout by consuming psychological resources and influencing the meaning system.

### 2.1 Definition of core concepts

#### 2.1.1 Academic burnout

Academic burnout is typically defined as a negative psychological state that develops in individuals during prolonged academic engagement, primarily characterized by emotional exhaustion, academic apathy, and diminished sense of accomplishment (Maslach et al., 2001). In academic contexts, academic burnout is viewed as a reactive outcome to sustained academic stress and resource depletion. This study employs academic burnout as a key outcome variable for measuring the academic adjustment status of junior college students.

#### 2.1.2 Perceived discrimination

Perceived discrimination refers to an individual’s subjective experience of unfair treatment based on their group identity within social interactions or institutional contexts (Schmitt et al., 2014). This concept emphasizes individuals’ subjective interpretations of unfair treatment rather than the objective discriminatory events themselves. In this study, perceived discrimination specifically refers to junior students’ subjective perceptions of unfair treatment based on their educational background, which is regarded as a source of social structural stress.

#### 2.1.3 Belief in a just world

Belief in a just world reflects an individual’s convictions about societal fairness and the link between effort and reward—that people generally get what they deserve (Furnham, 2003). It serves as a key part of one’s meaning system, helping to interpret social events and maintain a sense of predictability in the world. In this study, belief in a just world is used as a core indicator of the meaning system to explain how perceptions of discrimination lead to academic burnout.

#### 2.1.4 Sense of family obligation

Sense of family obligation refers to an individual’s internalization and identification with family role obligations (such as supporting the family, repaying parents, and assuming household responsibilities), reflecting the extent to which they regard family duties as essential requirements of their self-identity (Fuligni, 2001). This sense of responsibility manifests not only in behavioral commitment but also in psychological identification with family expectations and the meaning of responsibility. In this study, family responsibility is treated as a role-related individual difference variable to examine its moderating effect on the strength of the mediating pathway of perceived discrimination.

### 2.2 Theoretical framework

Based on the student needs-resources process framework, this study conceptualizes academic burnout as a process outcome that gradually emerges within the context of sustained learning demands and resource depletion. In educational stratification settings, perceived discrimination based on academic credentials can be viewed as a persistent social learning demand that may exacerbate imbalances between demands and resources during the learning process. Furthermore, from a meaning system perspective, perceived discrimination not only induces immediate emotional stress but may also undermine the foundational meaning sustaining long-term learning engagement by challenging individuals’ core beliefs regarding social fairness and the effort-reward connection. Among these, the belief in a just world—a crucial component of the meaning system—may mediate the relationship between perceived discrimination and academic burnout. Simultaneously, variations in individuals’ family role expectations—particularly differing levels of family responsibility—may influence how they interpret discriminatory experiences and allocate resources, thereby moderating the aforementioned causal pathways. Based on this, this study constructs an integrated theoretical model combining social stress, meaning systems, and role expectations to explain the psychological mechanisms through which perceived discrimination affects academic burnout among junior college students.

### 2.3 The role of perceived discrimination in academic burnout

Existing research generally views perceived discrimination as a persistent source of social stress and explains its impact on academic burnout within the “stress response-academic adjustment” framework. Researchers describe and compare experiences of discrimination, stress, and academic burnout within a unified framework, emphasizing that additional stress from discrimination often coexists with higher levels of burnout (Williams-York et al., 2024). In the Chinese context, studies targeting junior college students simultaneously measure perceived discrimination, negative emotions, and academic burnout. The pathway explanation “perceived discrimination—negative emotions—academic burnout” pathway to explain the impact of perceived discrimination on academic adaptation issues, reflecting a research orientation that integrates discrimination into the general psychological distress mechanism (Li et al., 2024). Beyond the “emotional distress—burnout” explanatory pathway, the resource perspective also provides important clues for understanding burnout formation. Based on models such as the demand-resource framework, burnout is often viewed as a process outcome that develops gradually under sustained demands and resource depletion. Academic burnout is not a singular, short-term emotion but is closely related to the resources available to individuals during the learning process and their depletion (Zhang et al., 2025). Concurrently, recent student burnout research emphasizes that burnout symptoms exhibit temporal development and individual differences (Turhan et al., 2023).

In summary, existing research consistently finds a significant correlation between perceived discrimination and academic burnout across diverse student populations, generally recognizing discrimination as a major social stressor affecting academic adjustment.

Therefore, this study proposes the following hypothesis: H1: Perceived discrimination significantly and positively predicts academic burnout among junior college students.

However, existing research offers limited explanatory depth for this relationship. First, some studies tend to treat discrimination as a generic stressor, primarily explaining its link to academic burnout through universal psychological distress variables such as negative emotions and stress (Li et al., 2024). Yet academic burnout is not merely a direct manifestation of stress or negative emotions; its core characteristics also encompass changes at the “motivation-meaning level,” such as withdrawal from learning, apathy, cynicism, and diminished efficacy (Madigan and Curran, 2021). Therefore, relying solely on generic mechanisms like stress or emotional distress may be insufficient to explain how perceived discrimination progressively erodes individuals’ long-term assessments of learning meaning, reward expectations, and rule fairness, ultimately leading to academic burnout. Based on this, it is necessary to further introduce meaning system variables that more closely align with the essence of burnout to reveal the more specific psychological mechanisms through which perceived discrimination influences academic burnout.

### 2.4 The mediating role of the just-world belief

#### 2.4.1 The influence of perceived discrimination on the just-world belief

From a meaning-system perspective, individuals do not passively react emotionally to experiences of discrimination. Instead, they understand the underlying social rules by processing the meaning of events (Park, 2010). Thus, perceived discrimination signifies not merely an “unpleasant encounter,” but may be interpreted as “rules are not fair to all” or “the connection between effort and reward is compromised,” thereby undermining individuals’ fundamental beliefs about social fairness and deservedness (Major and O’Brien, 2005). The just-world belief is a key component within this meaning system. When individuals are challenged by experiences of injustice, it further influences their adaptive strategies and behavioral manifestations (Furnham, 2003; Correia and Dalbert, 2007). Recent studies have begun examining “discrimination perception—just world belief” within the same model, exploring the underlying pathways. Therefore, among junior college students, discrimination perception may weaken their belief in a just world.

#### 2.4.2 The influence of fair world belief on academic burnout

Academic burnout is a psychological state that develops gradually under sustained learning demands, exhibiting temporal dynamics. Consequently, it is more likely linked to individuals’ long-term assessments of learning rewards, fairness, and controllability (Turhan et al., 2023). Research indicates that belief in a just world maintains a stable association with positive academic adaptation indicators (such as academic engagement) and may exert indirect effects through psychological resources like resilience. This provides empirical support for belief in a just world, as a core tenet of the meaning system, being linked to learning engagement and adaptation outcomes (Li et al., 2025). Research indicates that the belief in a just world maintains both direct and indirect relationships with learning satisfaction and learning engagement, further suggesting its foundational role in shaping the sense of value and academic commitment during the learning process. Therefore, when belief in a just world is low, maintaining learning engagement becomes more challenging, increasing susceptibility to academic burnout.

Based on the above analysis, we propose research hypothesis H2: Belief in a just world mediates the relationship between perceived discrimination and academic burnout. Specifically, Hypothesis H2a: Perceived discrimination significantly and negatively predicts belief in a just world. Hypothesis H2b: Belief in a just world significantly and negatively predicts academic burnout.

### 2.5 The moderating role of family responsibility

The impact of perceived discrimination on academic burnout does not affect all students with equal intensity. An individual’s role expectations may shape how they interpret and cope with stressful events. Existing research has found, across samples at different developmental stages, that psychological variables related to an individual’s role or identity can moderate the effect of perceived discrimination on adaptation outcomes. In a sample of migrant children, identity integration moderated the path from perceived discrimination to burnout, indicating that role expectation differences can alter the strength of this effect (Li et al., 2021). Meanwhile, family responsibility reflects the degree to which individuals internalize family role obligations, potentially altering resource allocation and stress thresholds between academic pursuits and familial duties (Nnubia and Eze, 2024). Research indicates systematic associations between college students’ family responsibilities—such as caregiving and financial obligations—and learning-related psychological variables like academic value and academic efficacy (Glover et al., 2025). This indicates that family responsibilities do not merely exist as external contextual factors but are embedded within individuals’ psychological evaluations of the meaning of learning commitment and the effort-reward relationship. Consequently, they exert a potential influence on learning persistence and exhaustion outcomes. Thus, family responsibility may function as a boundary condition, moderating the strength of discrimination perceptions’ effects on beliefs about learning meaning (e.g., fair world belief) and long-term learning adaptation outcomes (e.g., academic burnout).

However, existing research remains unclear on whether family responsibility buffers or exacerbates individuals’ burnout responses when encountering discrimination or other situations that may undermine learning meaning. On one hand, from the perspective of role expectations and meaning systems, family responsibility may exert a protective effect by endowing learning with a higher-order sense of responsibility. On the other hand, the accompanying persistent role obligations and responsibility pressures demand continuous investment of time and psychological energy. This long-term resource occupation may diminish an individual’s focus and regulatory capacity in learning contexts, thereby reducing the quality of learning engagement, accelerating resource depletion, and hastening the formation of burnout (Thoits, 1991). This study examines the moderating role of family responsibilities in a discrimination perception context, helping clarify their dual function in the learning adaptation process.

Therefore, we propose research hypothesis H3: Family obligations moderate the relationship between discrimination perception and academic burnout among junior college students.

The just-world belief reflects individuals’ fundamental understanding of social norms, fairness, and the effort-reward relationship, serving as a crucial component of the meaning system in learning contexts. Students with higher family responsibilities often view academic success as a vital pathway to fulfill family obligations and improve their family’s circumstances. Consequently, they are more sensitive to the belief that “effort should yield deserved rewards.” When such students perceive discrimination during their studies, they may be more inclined to interpret it as systemic injustice or a breakdown in the effort-reward connection, thereby inflicting a stronger impact on their belief in a just world. However, in certain contexts, the responsibility-driven motivation stemming from family obligations may also prompt individuals to maintain the belief that “effort is worthwhile” to sustain their commitment. Consequently, family responsibility may moderate the relationship between perceived discrimination and belief in a just world.

Based on this, we propose Hypothesis H4: Family obligations mediate the relationship between perceived discrimination and belief in a just world among junior college students.

**Figure 1:**
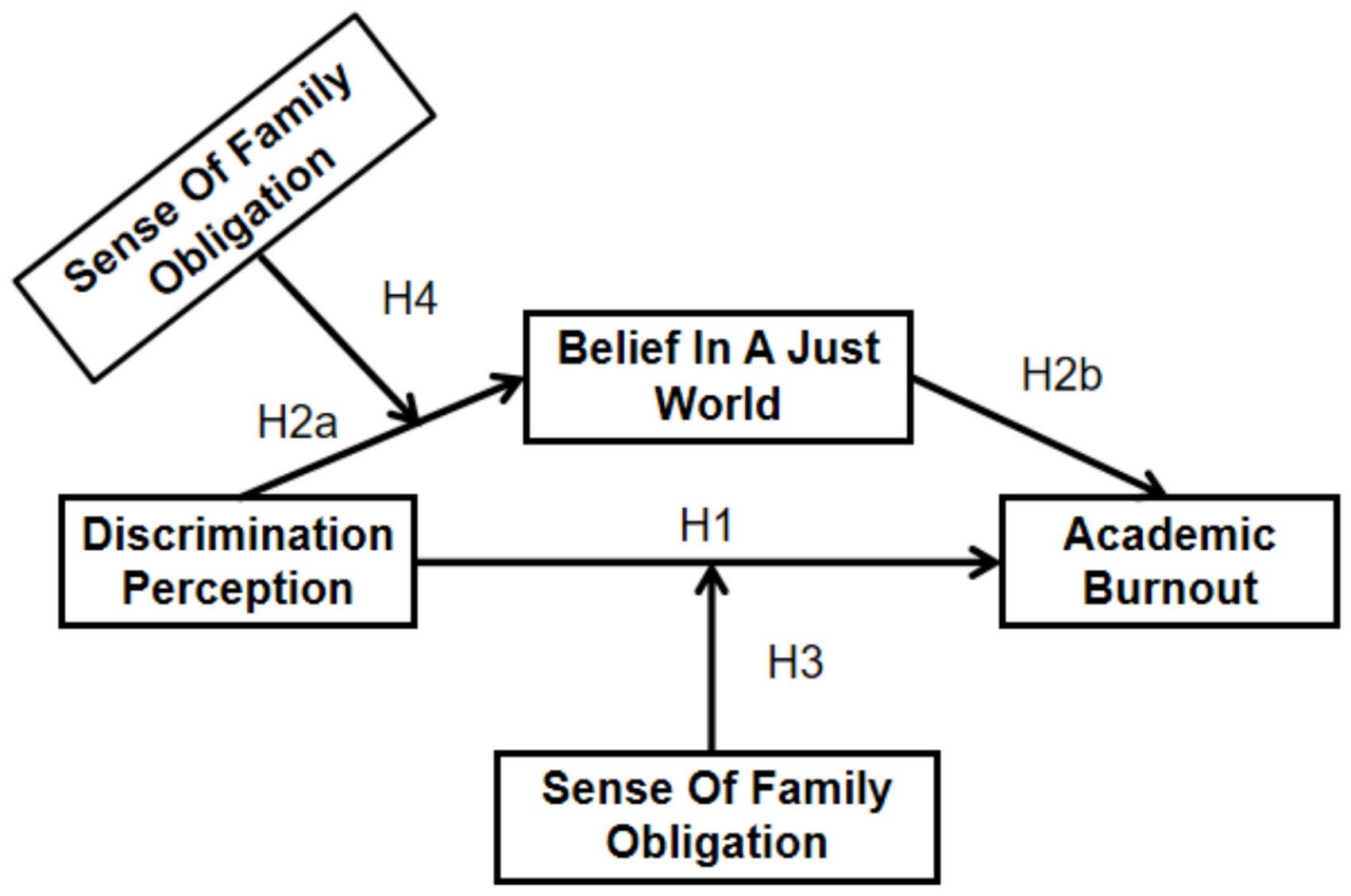
Hypothesis model.

In summary, this study will examine junior college students to investigate the mediating role of belief in a just world between perceived discrimination and academic burnout, as well as the moderating role of family obligations. The overall research hypotheses are presented in Figure 1.

## 3 Materials and methods

### 3.1 Participants and procedure

The data used in this paper are from a random sample survey of junior college students in Jinan. Since Jinan, as the provincial capital, can represent the general level of the developed provinces in eastern China and has certain representativeness nationwide, has a certain representativeness in the country, the research conclusions drawn from Jinan as a case area have certain reference value for exploring the academic burnout of junior college students in large and medium-sized cities across the country. At present, there are 18 junior colleges in Jinan. This study selected 18 junior college students in Jinan to conduct a random questionnaire survey.

The questionnaire was distributed and investigated by means of stratified sampling and random sampling. The principle of quotas is as follows. Firstly, within the city of Jinan, districts were taken as the primary sampling units. Five districts—Lixia District, Shizhong District, LiCheng District, Huaiyin District and Changqing District—were selected as sample survey areas based on their regional economic development status. Lixia District is the district with the highest economic development level in Jinan. Shizhong District, LiCheng District and Huaiyin District are at a medium level. Although Changqing District has a relatively weak economic development level, it is densely populated with colleges and universities. Therefore, two vocational colleges were randomly selected from each district in Jinan. Secondly, 150–200 junior college students were randomly selected from each sample college according to a certain proportion. The survey was conducted by distributing electronic questionnaires online. To ensure data quality, the online questionnaire requires students to complete it independently within the specified time through the designated link or QR code. In addition, during the questionnaire distribution process, the respondents were informed that this was an anonymous survey. After the data was collected, we strictly screened the questionnaires based on the following criteria to ensure the quality of the data. First, eliminate questionnaires whose answers show obvious regularity, such as linear or zigzag responses. Second, we will delete questionnaires with overly short response times. According to the pre-test, questionnaires with response times less than one-third of the average duration will be regarded as invalid. Third, questionnaires with more than 10% missing key scale questions were excluded. Finally, questionnaires with contradictory logic were eliminated. Therefore, a total of 1763 questionnaires were distributed and 1,499 valid questionnaires were collected. The age range of the junior college students participating in this study was mainly concentrated between 18 and 22 years old, with an average age of approximately 19.5 years old. The growth environment was that 816 people (54.4%) grew up in cities and 683 people (45.6%) grew up in rural areas. 209 people (20.6%) are only children, and 1,190 people (79.4%) are not only children. The descriptive statistical results of the sample are shown in Table 1.

**Table 1:** Sample descriptive statistics table.

Item	Category	Frequency	Percentage(%)
Sexuality	Males	303	20.2
	Female	1,196	79.8
Grade	Freshman	613	40.9
	Sophomore	713	47.6
	Junior	173	11.5
Profession	Liberal Arts	1,073	71.6
	Science	220	14.7
	Engineering Course	206	13.7
Growth environment	City	816	54.4
	Rural	683	45.6
Whether an only child	Yes	209	20.6
	No	1,190	79.4

### 3.2 Measures

#### 3.2.1 Junior college students’ discrimination perception questionnaire

By using the methods of literature analysis, interview, project analysis and confirmatory factor analysis, based on the “Migrant Children’s Discrimination Perception Questionnaire “compiled by Liu and Shen (2010), the “College Students’ Discrimination Perception Questionnaire” was compiled. A total of 16 items were designed in the questionnaire. After pre-investigation, items 3 and 4 with ambiguity of understanding were deleted, and the remaining 14 items included four dimensions: intelligence and performance discrimination, physical trait discrimination, language communication discrimination and family background discrimination. For example “Teachers like students with good grades”. The higher the score, the higher the level of discrimination perception of junior college students. After testing, the Cronbach’s *α* coefficient of the questionnaire was 0.938, and the KMO values were 0.954, indicating that the questionnaire had good reliability. Further tests were conducted on the revised questionnaire, the standardized factor loadings of all items in the questionnaire were > 0.50, the combined reliability of each sub-dimension was > 0.7, and the average variance draw was between 0.57 and 0.70. The CR of the overall scale is 0.901 and AVE is 0.695. All indicators met the metrological standards (CR > 0.7, AVE > 0.5), indicating that the scale and each of its sub-dimensions have good reliability and convergent validity (Table 2).

**Table 2:** The reliability and validity of the discrimination perception questionnaire.

Dimension	Item	Estimate	Cronbach’s α	CR	AVE
Intelligence and performance discrimination	A1	0.656	0.703	0.712	0.556
	A2	0.826			
Physical trait discrimination	B1	0.694	0.888	0.892	0.676
	B2	0.852			
	B3	0.836			
	B4	0.894			
language communication discrimination	C1	0.858	0.833	0.847	0.587
	C2	0.775			
	C3	0.527			
	C4	0.857			
family background discrimination	D1	0.787	0.901	0.904	0.704
	D2	0.918			
	D3	0.904			
	D4	0.733			
Total quantity table			0.938	0.901	0.695

#### 3.2.2 Junior college students’ academic burnout questionnaire

Based on the “Middle School Students’ Academic Burnout Questionnaire” (Hu and Dai, 2007) and the “College Students’ Academic Burnout Questionnaire” (Lian et al., 2005), this paper compiles a new “College Students’ Academic Burnout Questionnaire.” A total of 14 items were designed in the questionnaire, including four dimensions, learning inefficiency, depression, physiological exhaustion and academic alienation. For example “I don’t think I have the ability to get good grades”. Using the Likert’s five-point scoring method, the higher the score, the higher the level of academic burnout of junior college students. After testing, the Cronbach’s *α* coefficient of the questionnaire was 0.911 > 0.8, and the KMO values were 0.908 > 0.6, indicating that the questionnaire had good reliability.

#### 3.2.3 Junior college students’ belief in a just world questionnaire

Combined with the “Belief in a Just World Scale” (Dalbert, 1999) and the localization revision of this scale in China (Su et al., 2012), this paper takes the group of junior college students as the research object to modify, and the “College Students’ Belief in a Just World Questionnaire” was compiled. The questionnaire was designed with 13 items, including two dimensions general belief in a just world and personal belief in a just world. For example: “I think the society is basically fair”. Using Likert’s five-point scoring method, 1 represents “completely inconsistent” and 5 represents “completely consistent”. The higher the score, the higher the level of belief in a just world of junior college students. After testing, the Cronbach’s *α* coefficient of the questionnaire was 0.938>0.8, and the KMO values were 0.922 > 0.6, indicating that the questionnaire had good reliability.

#### 3.2.4 Junior college students’ sense of family obligation questionnaire

Combined with the questionnaire of the sense of family obligation compiled by Li (2017), the “College Students’ Sense of Family Obligation Questionnaire” revised by this study contains three dimensions, which are the current help to the family, the respect to the family and the support to the family in the future. For example, “take the initiative to do housework at home”. Using Likert’s five-point scoring method, 1 represents “completely inconsistent” and 5 represents “completely consistent”. The higher the score, the higher the sense of family obligation. After testing, the Cronbach’s α coefficient of the questionnaire was 0.963 > 0.8, and the KMO values were 0.963 > 0.6, indicating that the questionnaire had good reliability.

### 3.3 Data analysis

This paper uses SPSS23.0 and AMOS24.0 to analyze the internal relationship between discrimination perception, belief in a just world and academic burnout, and uses Bootstrap method to verify the mediating role of belief in a just world. The multiple hierarchical regression method was used to verify the moderating effect of the sense of family obligation between discrimination perception and academic burnout, discrimination perception and belief in a just world.

### 3.4 Common method deviation test

In this study, Harman’s single factor test method was used to conduct an unrotated exploratory factor analysis for all variables. The results showed that there were 10 common factors with characteristic roots greater than 1, and the variation explained by the first common factor was 30.58%, which was less than the critical value of 40%, indicating that there was no obvious common method bias in this study.

## 4 Results

### 4.1 Descriptive statistics

If the correlation coefficient is positive, and through the significance test, there is a significant positive correlation between the variables; if the correlation coefficient is negative, and through the significance test, there is a significant negative correlation between the variables. This study conducted a pairwise correlation analysis of discrimination perception, belief in a just world, academic burnout and sense of family obligation. The results are shown in Table 3.

**Table 3:** Correlation analysis.

Variable	Discrimination perception	Belief in a just world	Academic burnout	Sense of family obligation
Discrimination perception	1			
Belief in a just world	−0.274**	1		
Academic burnout	0.753**	−0.304**	1	
Sense of family obligation	−0.437**	0.386**	−0.442**	1

** refers to a significant correlation at the 0.01 level (two-tailed).

The table above is the result of correlation analysis. The results show that the *p* values corresponding to the correlation coefficients between discrimination perception, belief in a just world, academic burnout and sense of family obligation are all less than 0.01, which has significant statistical significance. It shows that there is a significant correlation between discrimination perception, belief in a just world, academic burnout and family obligation. The following structural equation model analysis can be performed.

### 4.2 Structural equation model test results

**Figure 2:**
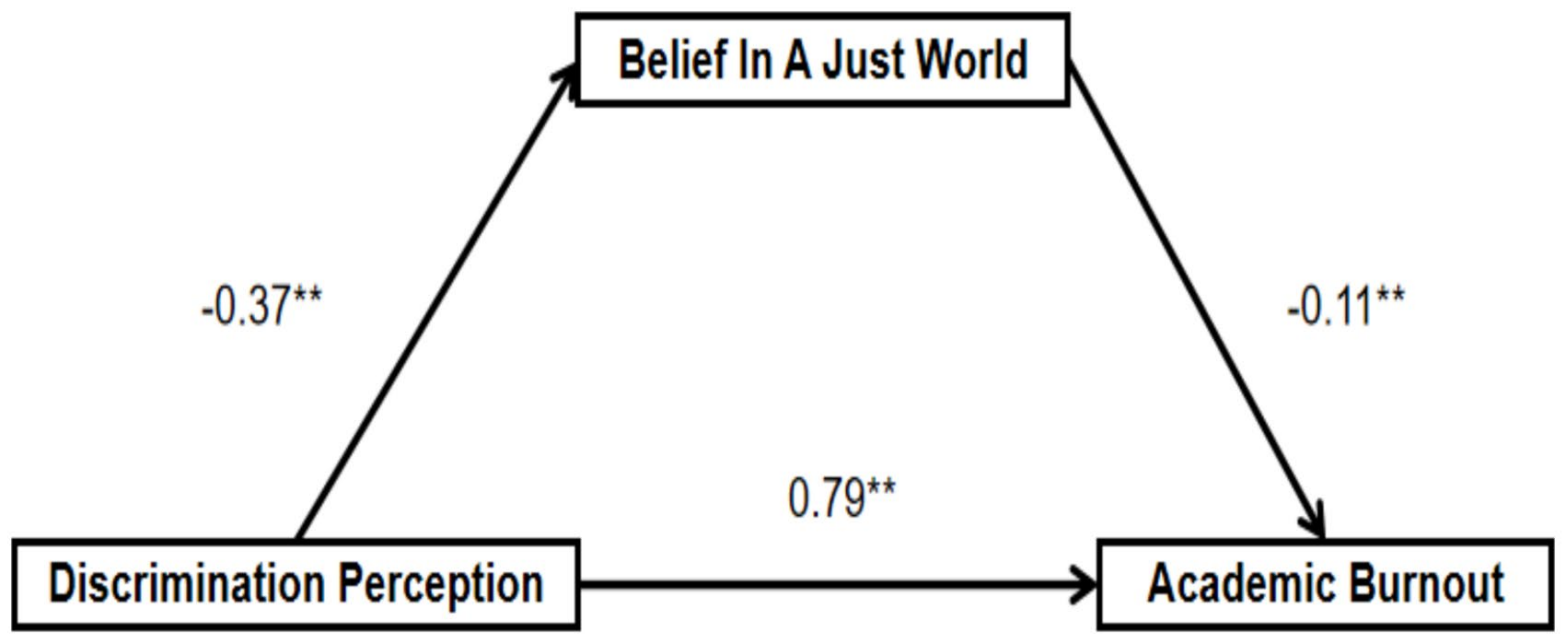
The mediating effect of belief in a just world in the relationship between discrimination perception and academic burnout. **p < 0.01.

The Bartlett’s Sphericity test results show that the significance probability corresponding to the approximate chi-square value is all 0.000 (*p* < 0.01). The total variance explanation rates of the four scales are 64.907, 67.764, 64.576, and 77.161% respectively, all greater than 60%. The load of each measurement item is higher than 0.5, and there is no situation where the double factor load is high. Moreover, the measurement items under each dimension are aggregated together according to the theoretical distribution. The above results indicate that the structural validity and content validity are good. The confirmatory factor analysis results show that the standardized factor load of each item is greater than 0.5, and the standard error value of S.E. is also less than the standard of 0.5, proving that the validity of the questionnaire is good. At the same time, the AVE of each dimension is greater than 0.5, and the square root of AVE is greater than the correlation coefficient between each variable, indicating that the variables of the scale have good convergent validity and discriminant validity. The fitting index of the structural equation model shows that the X^2^/DF value is 6.659, which is greater than 3. However, considering the large sample size, this paper holds that X^2^/DF less than 8 is acceptable. RMSEA = 0.061 < 0.08, indicating a good fit. GFI = 0.973, AGFI = 0.953, CFI = 0.980, TLI = 0.972 are all greater than the standard level of 0.9, and RMR = 0.016, indicating that the model fits well. Therefore, the structural equation model established in this study is effective. The result is shown in Table 4. AMOS results supported the hypotheses: discrimination perception significantly and positively predicted academic burnout (H1), while belief in a just world partially mediated this relationship (H2), with discrimination perception negatively predicting belief in a just world, which in turn negatively predicted academic burnout. Through the path analysis of the structural equation model, the path coefficient values and C.R values of the structural equation model were obtained, and the results are shown in Table 5 (see Figure 2).

**Table 4:** Overall fitting coefficient.

X^2^/DF	RMSEA	GFI	AGFI	CFI	TLI	IFI	RMR
6.659	0.061	0.973	0.953	0.980	0.972	0.980	0.016

**Table 5:** The path analysis results of the structural equation model.

Path	Unstd.	S.E.	C.R.	*P*
SJGZXN ← QSZJ	−0.371	0.032	−8.162	***
XYJD ← SJGZXN	−0.109	0.039	−4.575	***
XYJD ← QSZJ	0.790	0.031	29.243	***
Q14 ← QSZJ	0.870			
Q13 ← QSZJ	0.877	0.023	45.004	***
Q12 ← QSZJ	0.856	0.022	43.307	***
Q11 ← QSZJ	0.723	0.027	32.417	***
Q19 ← SJGZXN	0.543			
Q20 ← SJGZXN	0.839	0.119	9.061	***
Q15 ← XYJD	0.840			
Q16 ← XYJD	0.805	0.024	35.976	***
Q17 ← XYJD	0.726	0.033	31.679	***
Q18 ← XYJD	0.866	0.025	40.002	***

***p < 0.001.

### 4.3 The results of mediating effect analysis of belief in a just world

To control for the possible interference caused by demographic variables, this study fixed gender, grade, major, family, and whether they were the only child as control variables. The results showed that discrimination perception had a significant positive effect on academic burnout (*β* = 0.790, *p* < 0.01), while it negatively predicted belief in a just world (β = −0.371, *p* < 0.01). In turn, belief in a just world negatively predicted academic burnout (β = −0.109, *p* < 0.01).

The results of the mediating effect analysis in Table 6 indicate that discrimination perception affects academic burnout through the belief in a just world. By testing the mediating effect, it is found that the confidence interval does not include 0, which proves that the mediating effect is established, and the belief in a just world plays a partial mediating role between discrimination perception and academic burnout.

**Table 6:** Summary of mediating effect test results.

Effect relationship	Effect size	BootSE	95% bootstrap CI	Effect ratio
Gross effect	0.883**	0.0203	[0.843, 0.923]	
Direct effect	0.849**	0.0209	[0.808, 0.890]	96.07%
Indirect effect	0.035**	0.0076	[0.021, 0.051]	3.93%

**p < 0.01.

### 4.4 The analysis result of the moderating effect of sense of family obligation

**Figure 3:**
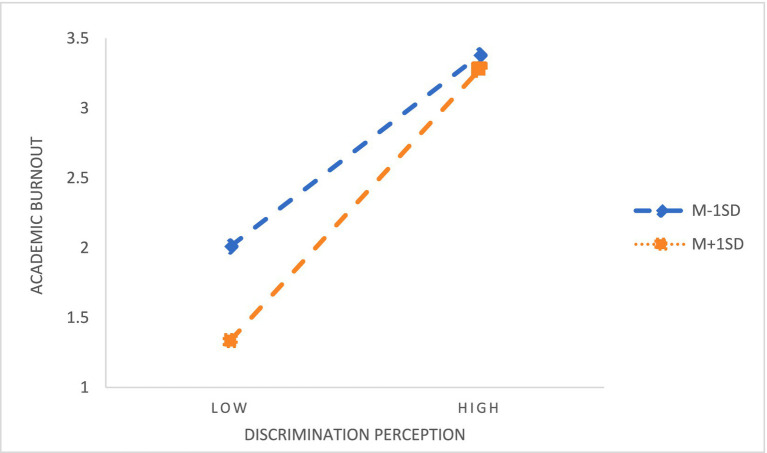
The moderating effect of family obligation between discrimination perception and academic burnout.

**Figure 4:**
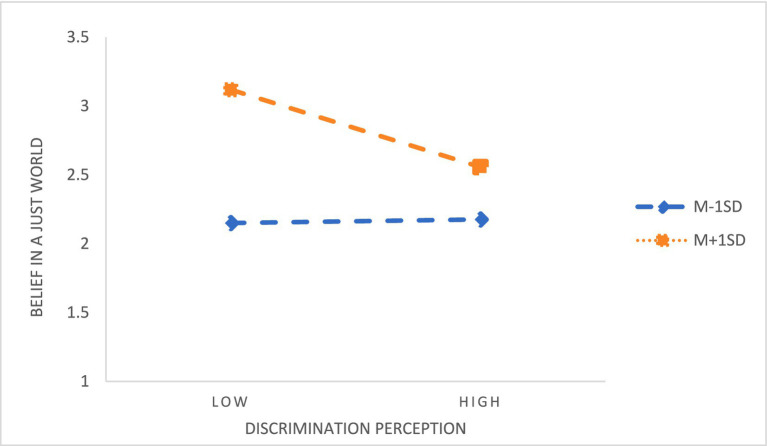
The moderating effect of family obligation between discrimination perception and belief in a just world.

Hierarchical regression results showed that sense of family obligation significantly predicted academic burnout (β = −0.140, *p* < 0.001) and belief in a just world (β = −0.335, *p* < 0.001), and its interaction with discrimination perception significantly predicted both outcomes (burnout: β = −0.085, *p* < 0.001; belief: β = −0.114, *p* < 0.001), supporting H3 and H4. After controlling for variables such as gender, grade, major, family status and whether one is an only child, the results are shown in Tables 7, 8. According to the standard of average plus or minus one standard deviation, the sense of family obligation was divided into two groups: high regulation and low regulation. The effects of discrimination perception on academic burnout (Figure 3) and the effects of discrimination perception on the belief in a just world (Figure 4) were investigated, respectively.

**Table 7:** The results of moderating effect analysis of family obligation (H3).

Variable	Model 1		Model 2		Model 3	
	β	t	β	t	β	t
Gender	−0.060*	−2.216	0.013	0.764	0.015	0.840
Grade	−0.056*	−2.164	−0.025	−1.467	−0.024	−1.412
Professional	−0.003	−0.124	0.006	0.325	0.008	0.460
Growth environment	0.128***	4.973	0.027	1.569	0.024	1.419
Whether an only child	0.014	0.535	−0.004	−0.211	−0.005	−0.311
Discrimination perception			0.688***	36.823	0.700***	37.423
Sense of family obligation			−0.140***	−7.469	−0.159***	−8.391
Discrimination perception × sense of family obligation					−0.085***	4.880

R^2^	0.024	0.585	0.591
△R^2^		0.561	0.007
F	7.254***	299.711***	269.237***

Dependent variable = academic burnout; * *p* < 0.05, *** *p* < 0.001.

**Table 8:** The results of moderating effect analysis of family obligation (H4).

Variable	Model 1		Model 2		Model 3	
	β	t	β	t	β	t
Gender	−0.011	−0.416	−0.058*	−2.331	−0.060*	−2.413
Grade	−0.006	−0.215	−0.005	−0.206	−0.006	−0.269
Professional	0.041	1.544	0.031	1.260	0.029	1.142
Growth environment	−0.047	−1.791	0.010	0.402	0.013	0.557
Whether an only child	−0.028	−1.055	−0.030	−1.218	−0.028	−1.152
Discrimination perception			0.132***	−5.007	−0.148***	−5.591
Sense of family obligation			−0.335***	12.691	0.362***	13.469
Discrimination perception × sense of family obligation					−0.114***	−4.626

R^2^	0.005	0.169	0.180
△R^2^		0.164	0.012
F	1.875	50.421***	46.905***

Dependent variable = belief in a just world; * *p* < 0.05, *** *p* < 0.001.

Simple slope analysis indicated that, regardless of obligation level, discrimination perception positively predicted burnout and negatively predicted belief in a just world; however, students with high family obligation reported overall lower burnout and higher belief. At very high levels of discrimination perception, burnout in the high-obligation group approached or exceeded that of the low-obligation group, while the negative impact of discrimination perception on belief in a just world was stronger under high obligation, confirming the moderating role of family obligation in both relationships.

## 5 Discussion

### 5.1 The relationship between perceived discrimination and academic burnout among junior college students

This study found that perceived discrimination significantly and positively predicted academic burnout among junior college students. This result aligns with the overall conclusions of existing research across different student populations regarding the relationship between “discrimination experiences/unfair treatment—burnout/exhaustion” (Teshome et al., 2022; Williams-York et al., 2024). Furthermore, this finding aligns with the fundamental expectation of the student demand-resource process framework adopted in this study—namely, that “persistently increasing learning demands heighten the risk of exhaustion and burnout.” This provides a process-oriented interpretive entry point for understanding the formation of academic burnout among junior college students, suggesting that academic burnout can be viewed as a process-oriented outcome that gradually develops when learning demands accumulate continuously during sustained academic engagement, thereby persistently depleting available resources (Lesener et al., 2020; Demerouti et al., 2001).

Unlike some studies that focus more on discrimination’s impact on immediate emotional responses or general stress experiences, this research emphasizes that in the context of higher education stratification and academic credential labeling: For junior college students, perceived discrimination based on educational identity can be conceptualized as an ongoing demand for social learning—not a one-time negative experience, but one that may be repeatedly activated in academic evaluations, interpersonal interactions, and expectations of future opportunities, thereby forming an inescapable and cumulative psychological burden. This interpretation of discrimination as chronic stress exposure with cumulative effects aligns with meta-analytic evidence (Pascoe and Smart Richman, 2009). From a demand-resource perspective, when such social evaluation pressures persist long-term and exceed students’ available psychological resources (e.g., emotional regulation capacity, sense of belonging, and locus of control), resources become continuously occupied and depleted. This deepens the demand-resource imbalance, thereby increasing susceptibility to burnout. Consistent with this chain of processes, the present study found that perceived discrimination exerted a strong direct predictive effect on academic burnout (*β* = 0.790). This suggests that perceived discrimination, as a form of social evaluation stress, may be closely linked to the sustained depletion of psychological resources required to maintain academic engagement. Furthermore, commonly cited reactions in existing research—such as anxiety, depression, diminished self-efficacy, and avoidant coping—can be understood as manifesting forms of “persistent resource depletion” at different levels. For instance, to avoid further negative evaluations and risks of identity exposure, students may reduce classroom interaction or lower learning engagement for short-term relief, but this long-term weakens engagement quality and accelerates burnout accumulation.

The theoretical advancement of this finding lies in expanding the research perspective on academic burnout from individual internal factors (such as personality traits) or immediate learning context pressures to persistent social structural pressures. It further extends to persistent structural pressures linked to students’ social evaluation environments and institutional contexts. This implies that academic burnout among junior college students is not merely an individual issue of “being unable to concentrate” or “feeling down,” but to a significant extent reflects their structural position within the educational ecosystem and social evaluation system. Therefore, to comprehensively understand and intervene in academic burnout, it must be examined within the context of social stratification and educational equity. The social evaluation environment—such as differential treatment stemming from academic credential labeling—should be incorporated into the analytical framework as a key exogenous learning demand. Simultaneously, given that perceived discrimination often touches upon foundational learning meanings such as “fair evaluation/effort-reward linkage,” subsequent analyses will further examine its mechanisms and boundary conditions from a meaning system perspective (see 5.2 and 5.3).

### 5.2 Mediating role of the just-world belief

This study finds that the belief in a just world partially mediates the relationship between perceived discrimination and academic burnout. It reveals that perceived discrimination influences academic burnout not only through relatively superficial pathways such as “persistent resource depletion and negative emotional reactions,” but also by undermining the meaning system that underpins sustained learning engagement. In other words, this study advances the explanation of the discrimination perception-burnout relationship from a single “stress-response/resource depletion” pathway to a deeper psychological mechanism involving “damage to foundational meaning.” This provides a new perspective for understanding why academic burnout among junior college students exhibits cumulative and chronic characteristics (Maslach and Leiter, 2016; Salmela-Aro et al., 2020).

From the perspective of the meaning system, the impact of perceived discrimination on junior college students extends far beyond triggering immediate discomfort or stress; it fundamentally alters students’ interpretive frameworks for societal norms. Compared to the demand-resource model, which focuses more on explaining “how external pressures consume individual resources and lead to exhaustion” (Demerouti et al., 2001), the meaning system approach pays greater attention to how repeated experiences of unfair treatment based on educational background cause students to question fundamental beliefs such as “whether effort will be rewarded” and “whether social rules are fair.” In this study, perceived discrimination significantly and negatively predicted belief in a just world (*β* = −0.371), confirming this erosion of the meaning system. As a core belief underpinning individuals’ understanding of societal norms and their expectation of future controllability, the undermining of belief in a just world directly diminishes the sense of meaning and purpose in learning (Lipkusa et al., 1996). When students no longer firmly believe that “diligent study can change one’s destiny” or that “effort will receive fair evaluation,” the intrinsic motivation to sustain long-term, high-commitment academic engagement will diminish. This is precisely the underlying logic behind the negative prediction of academic burnout by the belief in a just world (β = −0.109).

Therefore, the theoretical contribution of this study lies in integrating the demand-resource framework with the meaning system perspective to propose a dual-pathway model. Perceived discrimination leads to burnout both directly by depleting psychological resources (the demand-resource pathway) and indirectly by eroding the “foundation of meaning” (fair world belief) that underpins sustained learning engagement. This explains why academic burnout constitutes a “chronic” state—it stems not only from immediate resource depletion but also from the gradual corrosion of the meaning system (May et al., 2015). This finding advances the understanding of academic burnout’s formation mechanism beyond the relatively superficial “stress-response” model to a deeper level concerning individual value beliefs and expectations of future controllability.

### 5.3 Moderating role of family obligation

Research findings indicate that a sense of family obligation exerts a significant negative moderating effect on both the relationship between perceived discrimination and academic burnout, as well as the relationship between perceived discrimination and belief in a just world. To interpret these results meaningfully, they must be contextualized within China’s unique family culture and educational traditions, thereby enabling a deeper understanding of the underlying psychological mechanisms.

Within mainstream Western psychological frameworks, family support is typically conceptualized as an external resource whose primary mechanisms involve buffering stress and providing emotional comfort (House et al., 1988). This perspective, rooted in individualistic cultures, emphasizes the role of family as a “source of support” that facilitates individuals’ pursuit of independent goals (Markus and Kitayama, 1991). However, within China’s collectivist culture, the concept of familial obligation carries greater complexity—it extends beyond receiving external support to encompass an internalized sense of responsibility and meaning system (Fuligni et al., 1999). Values such as filial piety and bringing honor to one’s ancestors make family obligations a core component of the individual’s self-concept (Yeh, 2009). Therefore, understanding the role of family obligations among Chinese students requires moving beyond the Western “social support-stress buffer” model to focus on its unique function as an internal meaning system and source of motivation.

For Chinese students, the sense of family obligation is not merely a psychological burden; it simultaneously carries higher-order learning significance (Tseng, 2004). For junior college students with a strong sense of family obligation, academic success is imbued with value that transcends personal achievement—it serves as a means to fulfill responsibilities to the family, repay parental sacrifices, and achieve upward mobility for the household. This sense of mission—“studying for the family”—provides a protective buffer against the impact of discrimination (Park, 2010). Even when faced with unfair external evaluations (perceived discrimination), the meaning of learning remains partially intact due to its binding to familial responsibility. This buffers the direct impact of discrimination on academic commitment (weakening the predictive power of perceived discrimination on burnout).

Simultaneously, a sense of family obligation moderates the erosion of belief in a just world caused by discrimination. For students with a strong sense of family obligation, their belief in whether the world is fair is closely tied to their ability to fulfill family responsibilities through effort. When confronted with discrimination, they may be more inclined to interpret injustice as temporary, external obstacles rather than a fundamental negation of the “effort-reward” principle (Lucas et al., 2011). This drives them to work harder to maintain the belief that “the world is fundamentally fair” in order to sustain their fulfillment of responsibilities. This explains why, under high family obligation, the negative impact of perceived discrimination on the belief in a just world is mitigated.

However, this moderating effect also reveals the dual nature of family obligation (Telzer and Fuligni, 2009). It serves both as a protective factor (providing meaning and buffering stress) and, under extreme conditions, as a stress amplifier. This study found that when perceived discrimination levels were extremely high, the burnout levels in the high family obligation group approached or even exceeded those in the low obligation group. This may occur because when discrimination becomes severe enough to undermine the fundamental pathway of “fulfilling family responsibilities through academic achievement,” individuals with high obligation experience intensified feelings of failure and pressure from “letting their family down,” which paradoxically accelerates burnout. This reflects the dual nature of ‘responsibility’ and “pressure” within Chinese family culture.

The theoretical contribution of this study lies in repositioning the sense of family obligation from an “external support resource” within Western frameworks to an intrinsically meaningful system with complex motivational dynamics within the Chinese cultural context (Hou et al., 2020). This paradigm shift not only illuminates the positive role of familial support in countering social discrimination but also cautions against the risks of overly internalized family responsibilities under extreme stress. It offers a crucial cultural-psychological lens for understanding the unique aspects of Chinese students’ academic adaptation. More significantly, this study challenges the direct application of Western theories to Chinese contexts, emphasizing the central role of culturally specific mechanisms in psychological processes.

### 5.4 Implications for educational practice

Based on the conclusions of this study, future interventions targeting academic burnout among college students should focus on the following aspects: (1) integrating cognitive-behavioral techniques to address maladaptive perfectionism (as indicated by our data), with specific modules including cognitive restructuring (e.g., challenging “all-or-nothing” thinking), behavioral experiments (e.g., controlled failure tasks), and incorporating mindfulness training to alleviate anxiety; (2) establishing a structured peer-support system within academic settings to mitigate emotional exhaustion, implemented through multi-tiered networks (e.g., trained “Academic Buddies” and ethically-guided peer listening groups), digital matching platforms, and sustainable mechanisms linked to academic credits; (3) tailoring strategies to discipline-specific stressors (e.g., addressing project-based workloads in STEM fields through collaboration training and normalization of setbacks, or alleviating writing anxiety in humanities through flexible writing workshops). These intervention measures provide practical value for campus mental health services. Institutions can adopt a phased implementation pathway, starting with pilot interventions in specific departments, gradually integrating the above intervention plans into mandatory mental health courses, and ultimately incorporating them into institutional quality assessment systems to translate research findings into sustainable practice. Future research should validate the effectiveness of these intervention plans through longitudinal intervention experiments.

This study has several limitations that should be acknowledged. First, the sample exhibited imbalances in gender distribution (higher proportion of female participants) and academic disciplines (overrepresentation of humanities majors), which may limit the generalizability of findings across genders and fields of study. Second, the cross-sectional design precludes causal inferences about the relationships between observed variables. Third, data were collected solely from Jinan, the capital city of Shandong Province—a region historically influenced by Confucian culture where familial expectations may be more conservative compared to other Chinese regions. This geographical and cultural specificity may affect the transferability of results to populations in more diverse socioeconomic or cultural settings.

Future research should address these limitations by: (1) recruiting gender-balanced and discipline-diverse samples to enhance representativeness; (2) employing longitudinal or experimental designs to examine causal pathways in academic burnout development; and (3) expanding data collection to multiple regions (such as coastal and inland, urban and rural) to assess the moderating role of cultural and socioeconomic factors. Comparative studies across different educational systems (such as vocational and comprehensive universities) would also provide deeper insights into context-specific mechanisms.

## Data Availability

The datasets presented in this study can be found in online repositories. The names of the repository/repositories and accession number(s) can be found in the article/supplementary material.

## References

[ref1] Bakker A. B. Mostert K. (2024). Study demands-resources theory: understanding student well-being in higher education. Educ. Psychol. Rev. 36. doi: 10.1007/s10648-024-09940-8

[ref2] Bask M. Salmela-Aro K. (2013). Burned out to drop out: exploring the relationship between school burnout and school dropout. Eur. J. Psychol. Educ. 28, 511–528. doi: 10.1007/s10212-012-0126-5

[ref3] China Youth Net. (2025). *E. coli*. Available online at: https://www.youth.cn (Accessed April 15, 2025).

[ref4] Correia I. Dalbert C. (2007). Belief in a just world, justice concerns, and well-being at Portuguese schools. Eur. J. Psychol. Educ. 22:421. doi: 10.1007/BF03173464

[ref9008] Dalbert C. (1999). The world is more just for me than generally: about the personal belief in a just world scale’s validity. Soc. Justice Res 12, 79–98. doi: 10.1023/A:1022091609047

[ref5] Demerouti E. Bakker A. B. Nachreiner F. Schaufeli W. B. (2001). The job demands-resources model of burnout. J. Appl. Psychol. 86, 499–512. doi: 10.1037/0021-9010.86.3.499 11419809

[ref9007] Fajardo-Bullón F. Pérez-Mayo J. Esnaola I. Anderson I. Knutagård M. (2020). Influence of psychosocial variables on the health of people living in housing exclusion. Int. J. Environ. Res. Public Health, 17, 8983. doi: 10.3390/ijerph17238983 33276637 PMC7730692

[ref6] Fuligni A. J. (2001). Family obligation and the academic motivation of adolescents from Asian, Latin American, and European backgrounds. New Dir. Child Adolesc. Dev. 94, 61–75. doi: 10.1002/cd.31 11873482

[ref7] Fuligni A. J. Tseng V. Lam M. (1999). Attitudes toward family obligations among American adolescents with Asian, Latin American, and European backgrounds. Child Dev. 70, 1030–1044. doi: 10.1111/1467-8624.00075

[ref8] Furnham A. (2003). Belief in a just world: research progress over the past decade. Personal. Individ. Differ. 34, 795–817. doi: 10.1016/s0191-8869(02)00072-7

[ref9] Glover C. S. Bámaca M. Y. Homma K. (2025). Do family obligations contribute to academic values? The mediating role of academic efficacy. Behav. Sci. 15. doi: 10.3390/bs15091212, 41009241 PMC12466623

[ref10] Goode W. J. (1960). A theory of role strain. Am. Sociol. Rev. 25, 483–496. doi: 10.2307/2092933

[ref11] Hou Y. B. Peng K. P. Zhu Y. (2020). Cultural psychology. Beijing: Peking University Press.

[ref12] House J. S. Umberson D. Landis K. R. (1988). Structures and processes of social support. Annu. Rev. Sociol. 14, 293–318. doi: 10.1146/annurev.so.14.080188.001453

[ref9005] Hu Q. Dai C. (2007). Research on the structure of middle school students’ academic burnout. Psychological Science. 30, 162–164. doi: 10.16719/j.cnki.1671-6981.2007.01.041

[ref13] Jin M. Zeng L. Zeng X. Huang Y. (2022). Mindfulness and learning burnout in high school students: a perspective based on self-regulated learning model. Stud. Psychol. Behav. 20, 494–500. doi: 10.12139/j.1672-0628.2022.04.009

[ref14] Lerner M. J. (1980). The belief in a just world: A fundamental delusion. New York: Plenum Press: Internet Archive.

[ref15] Lesener T. Gusy B. Wolter C. (2020). The job demands-resources model: a meta-analytic review of longitudinal studies. Work Stress 34, 76–103. doi: 10.1080/02678373.2018.1529065

[ref9002] Lian R. Yang L. Wu L. (2005). The relationship between professional commitment and learning burnout of junior college students and the development of the scale. Psychol. J. 29, 47–51. doi : 10.16719/j.cnki.1671-6981.2006.01.013

[ref9003] Li M. (2017). Research on the relationship between college students’ sense of family obligation, parents’ expectations and future orientation. Master’s thesis, Nanjing Normal University.

[ref16] Li J. Bai J. Q. Ouyang L. X. Lin H. (2025). How belief in a just world shapes academic engagement among Chinese college art majors: a cross-level moderated mediation model. PLoS One 20. doi: 10.1371/journal.pone.0317583, 39841666 PMC11753692

[ref17] Li W. P. Xu T. Diao L. T. Wu Q. S. (2024). The impact of perceived discrimination on Mobile phone addiction among Chinese higher vocational college students: a chain mediating role of negative emotions and learning burnout. Psychol. Res. Behav. Manag. 17, 401–411. doi: 10.2147/prbm.S440958 38343428 PMC10854227

[ref18] Li J. Zhao Y. H. Dong S. H. (2021). The effects of individual- and group-based perceived discrimination on academic burnout among migrant children: the mediating role of academic self-handicapping and the moderating role of identity integration. J. Psychol. Sci. 44, 1111–1118. doi: 10.16719/j.cnki.1671-6981.20210512

[ref19] Lipkusa I. M. Dalbert C. Siegler I. C. (1996). The importance of distinguishing the belief in a just world for self versus for others: implications for psychological well-being. Personal. Soc. Psychol. Bull. 22, 666–677. doi: 10.1177/0146167296227002

[ref9001] Liu X. Shen J. (2010). Migrant children’s perceived discrimination and its relationship with self-esteem. Psychol. Sci. 33, 695–697. doi: 10.16719/j.cnki.1671-6981.2010.03.044

[ref20] Lucas T. Zhdanova L. Alexander S. (2011). Procedural and distributive justice beliefs for self and others: assessment of a four-factor individual differences model. J. Individ. Differ. 32, 14–25. doi: 10.1027/1614-0001/a000032

[ref21] Madigan D. J. Curran T. (2021). Does burnout affect academic achievement? A Meta-analysis of over 100,000 students. Educ. Psychol. Rev. 33, 387–405. doi: 10.1007/s10648-020-09533-1

[ref22] Major B. O’Brien L. T. (2005). The social psychology of stigma. Annu. Rev. Psychol. 56, 393–421. doi: 10.1146/annurev.psych.56.091103.070137 15709941

[ref23] Markus H. R. Kitayama S. (1991). Culture and the self: implications for cognition, emotion, and motivation. Psychol. Rev. 98, 224–253. doi: 10.1037/0033-295X.98.2.224

[ref24] Maslach C. Leiter M. P. (2016). Understanding the burnout experience: recent research and its implications for psychiatry. World Psychiatry 15, 103–111. doi: 10.1002/wps.20311, 27265691 PMC4911781

[ref25] Maslach C. Schaufeli W. B. Leiter M. P. (2001). Job burnout. Annu. Rev. Psychol. 52, 397–422. doi: 10.1146/annurev.psych.52.1.397 11148311

[ref9009] May R. W. Bauer K. N. Fincham F. D. (2015). School burnout: diminished academic and cognitive performance. Learn. Individ. Differ. 42, 126–131. doi: 10.1016/j.lindif.2015.07.015

[ref27] Ministry of Education of the People’s Republic of China (2022). Development report of vocational education in China (2012–2022). Beijing: Ministry of Education.

[ref28] Ministry of Education of the People’s Republic of China. (2025). *E. coli*. Available online at: http://www.moe.gov.cn/jyb_sjzl/sjzl_fztjgb/202506/t20250611_1193760.html (Accessed June 18, 2025).

[ref29] Nnubia U. I. Eze F. E. (2024). Exploring family and academic role conflict among undergraduate students of the University of Nigeria. High. Educ. 88, 661–681. doi: 10.1007/s10734-023-01137-2

[ref30] Park C. L. (2010). Making sense of the meaning literature: an integrative review of meaning making and its effects on adjustment to stressful life events. Psychol. Bull. 136, 257–301. doi: 10.1037/a0018301, 20192563

[ref31] Pascoe E. A. Smart Richman L. (2009). Perceived discrimination and health: a meta-analytic review. Psychol. Bull. 135, 531–554. doi: 10.1037/a0016059, 19586161 PMC2747726

[ref32] Salmela-Aro K. Kiuru N. Leskinen E. Nurmi J.-E. (2009). School burnout inventory: reliability and validity. Eur. J. Psychol. Assess. 25, 48–57. doi: 10.1027/1015-5759.25.1.48

[ref33] Salmela-Aro K. Upadyaya K. (2014). School burnout and engagement in the context of demands-resources model. Br. J. Educ. Psychol. 84, 137–151. doi: 10.1111/bjep.12018, 24547758

[ref9006] Salmela-Aro K. Upadyaya K. (2020). School engagement and school burnout profiles during high school – the role of socio-emotional skills. Eur. J. Dev. Psychol., 17, 943–964. doi: 10.1080/17405629.2020.1785860

[ref34] Salmela-Aro K. Upadyaya K. Hietajärvi L. (2022). School engagement and burnout profiles during high school: a longitudinal study of adolescents in Finland. Educ. Psychol. 42, 439–456. doi: 10.1080/01443410.2021.2022532

[ref35] Schmitt M. T. Branscombe N. R. Postmes T. Garcia A. (2014). The consequences of perceived discrimination for psychological well-being: a Meta-analytic review. Psychol. Bull. 140, 921–948. doi: 10.1037/a0035754, 24547896

[ref9004] Su Z. Zhang D. Wang X. (2012). Revision of the belief in a just world scale and its reliability and validity in junior college students. Chin. J. Behav. Med. Brain Sci. 21, 561–563. doi: 10.3760/cma.j.issn.1674-6554.2012.06.026

[ref36] Telzer E. H. Fuligni A. J. (2009). Daily family assistance and the psychological well-being of adolescents from Latin American, Asian, and European backgrounds. Dev. Psychol. 45, 1177–1189. doi: 10.1037/a0014728, 19586187

[ref37] Teshome A. Andualem M. Girmay A. (2022). Perceived discrimination and burnout among university students: the mediating role of self-esteem. BMC. Psychology 10:156. doi: 10.1186/s40359-022-00863-2

[ref38] Thoits P. A. (1991). On merging identity theory and stress research. Soc. Psychol. Q. 54, 101–112. doi: 10.2307/2786929

[ref39] Tseng V. (2004). Family interdependence and academic adjustment in college: youth from immigrant and U.S.-born families. Child Dev. 75, 966–983. doi: 10.1111/j.1467-8624.2004.00717.x, 15144497

[ref40] Turhan D. Scheunemann A. Schnettler T. Bäulke L. Thies D. O. Dresel M. . (2023). Temporal development of student burnout symptoms: sociodemographic differences and linkage to university dropout intentions. Contemp. Educ. Psychol. 73. doi: 10.1016/j.cedpsych.2023.102185

[ref41] Valentino K. Park I. J. K. Cruz-Gonzalez M. Zhen-Duan J. Wang L. Yip T. . (2025). Family-level moderators of daily associations between discrimination and distress among Mexican-origin youth. Dev. Psychopathol. 37, 902–917. doi: 10.1017/S0954579424000749, 38584283 PMC11458824

[ref43] Williams-York B. Guenther G. A. Patterson D. G. Mohammed S. A. Kett P. M. Dahal A. . (2024). Burnout, exhaustion, experiences of discrimination, and stress among underrepresented and first-generation college students in graduate health profession education. Phys. Ther. 104. doi: 10.1093/ptj/pzae095, 39018222

[ref44] Yeh G. H. (2009). The dual filial piety model: retrospect and prospect. Indig. Psychol. Res. Chin. Soc. 32, 101–148.

[ref46] Zhang J. Meng J. W. Wen X. (2025). The relationship between stress and academic burnout in college students: evidence from longitudinal data on indirect effects. Front. Psychol. 16. doi: 10.3389/fpsyg.2025.1517920, 40491945 PMC12146318

